# B-Cell Homeostasis Is Maintained During Two Months of Head-Down Tilt Bed Rest With or Without Antioxidant Supplementation

**DOI:** 10.3389/fimmu.2022.830662

**Published:** 2022-02-16

**Authors:** Julie Bonnefoy, Bjorn Baselet, Dominique Moser, Stéphanie Ghislin, Silvana Miranda, Elodie Riant, Randy Vermeesen, Annekathrin M. Keiler, Sarah Baatout, Alexander Choukér, Jean-Pol Frippiat

**Affiliations:** ^1^ Stress Immunity Pathogens Laboratory, UR7300 SIMPA, Faculty of Medicine, Université de Lorraine, Vandoeuvre-lès-Nancy, France; ^2^ Radiobiology Unit, Institute for Environment, Health and Safety, Belgian Nuclear Research Center (SCK CEN), Mol, Belgium; ^3^ Laboratory of Translational Research Stress and Immunity, Department of Anesthesiology, Hospital of the Ludwig-Maximilians-University (LUM), Munich, Germany; ^4^ Cytometry Facility, I2MC, Université de Toulouse, Inserm, Université Toulouse III – Paul Sabatier (UPS), TRI Genotoul, Toulouse, France; ^5^ Institute of Doping Analysis and Sports Biochemistry Dresden, Kreischa, Germany; ^6^ Department of Molecular Biotechnology, Faculty of Biosciences Engineering, Ghent University, Ghent, Belgium

**Keywords:** bed rest, spaceflight, immune system, B-cell, antibody isotypes, inflammation, bone, antioxidant supplementation

## Abstract

Alterations of the immune system could seriously impair the ability to combat infections during future long-duration space missions. However, little is known about the effects of spaceflight on the B-cell compartment. Given the limited access to astronaut samples, we addressed this question using blood samples collected from 20 healthy male volunteers subjected to long-duration bed rest, an Earth-based analog of spaceflight. Hematopoietic progenitors, white blood cells, total lymphocytes and B-cells, four B-cell subsets, immunoglobulin isotypes, six cytokines involved in inflammation, cortisone and cortisol were quantified at five time points. Tibia microarchitecture was also studied. Moreover, we investigated the efficiency of antioxidant supplementation with a cocktail including polyphenols, omega 3, vitamin E and selenium. Our results show that circulating hematopoietic progenitors, white blood cells, total lymphocytes and B-cells, and B-cell subsets were not affected by bed rest. Cytokine quantification suggested a lower systemic inflammatory status, supported by an increase in serum cortisone, during bed rest. These data confirm the *in vivo* hormonal dysregulation of immunity observed in astronauts and show that bed rest does not alter B-cell homeostasis. This lack of an impact of long-term bed rest on B-cell homeostasis can, at least partially, be explained by limited bone remodeling. None of the evaluated parameters were affected by the administration of the antioxidant supplement. The non-effectiveness of the supplement may be because the diet provided to the non-supplemented and supplemented volunteers already contained sufficient antioxidants. Given the limitations of this model, further studies will be required to determine whether B-cell homeostasis is affected, especially during future deep-space exploration missions that will be of unprecedented durations.

## Introduction

Bioastronautics programs have substantially expanded over the last 50 years. Medical and physiological findings from these missions have demonstrated that spaceflight negatively impacts numerous physiological systems. Spaceflight causes muscle atrophy, bone demineralization, cardiovascular and metabolic dysfunctions, impaired cognitive processes and reduced immunological competence, which can all affect crew health and performance.

Previous studies regarding spaceflight-induced immune dysfunctions have mainly focused on innate immunity and T-cell responses [reviewed in (1, 2)], while humoral immunity, despite its important functions, has rarely been investigated (3). Space studies performed using the newt *Pleurodeles waltl* as an animal model showed that spaceflight affects antibody production in response to antigenic stimulation (4, 5) and that somatic hypermutations, which diversify antibody binding sites to improve their affinity, occur at a lower frequency in space following immunization (6). These data show that spaceflight quantitatively and qualitatively affects the amphibian humoral immune response and are translatable to mammals because the cardinal elements of the adaptive immune system are shared by all gnathostomes (7, 8). Indeed, two recent studies (9, 10) revealed some changes in antibody gene segment usage in immunized mice subjected to four weeks of anti-orthostatic suspension (AOS), a model commonly used to mimic physiological changes observed during space missions (11). Furthermore, another study showed that two out of five cosmonauts involved in a long-term space mission onboard the International Space Station (ISS) presented significant changes in their IgM repertoire during the mission, that these modifications persisted up to 30 days after landing and likely affected the specificities of IgM binding sites (12). This last observation agrees with another study that demonstrated that the immune system of approximately half of astronauts who spent six months on the ISS is sensitive to spaceflight conditions (13).

Regarding the production of B-cells, altered transcription levels of IgM heavy chains and of the lymphoid-determining transcription factor Ikaros were observed in *P. waltl* embryos subjected to gravity changes (14), thereby bringing to light a potential alteration in B lymphopoiesis that was later demonstrated in mice subjected to 21 days of AOS. This treatment induced progressive changes in bone structure and a reduction in common lymphoid progenitors and cells at the pro-B, pre-B and immature B stages in femoral bone marrow (15). In the same way, a proteomic study of the femurs of mice flown for one month onboard the BION-M1 biosatellite revealed a statistically significant decrease in the expression of several proteins required for the development of immune and bone cells, and flow cytometry analyses revealed 1.8-2.5-fold reductions in B-cells in the bone marrow and spleen one week after landing (16). On the other hand, a recent analysis of eight crewmembers who stayed 6 months onboard the ISS found that spaceflight does not seem to have a major impact on the number and proportion of circulating B-cell subsets (17). In contrast, another recent study highlighted a significant increase in B-cell percentage on landing day in 5 cosmonauts who stayed for the same duration on the ISS (12). This increase was transient, as B-cells returned to baseline levels seven days after landing. These discrepancies among the organisms studied, and also between studies conducted in humans, indicate an urgent need for further research to determine whether B-cell homeostasis is affected in response to long-duration spaceflight.

To address this question and given limitations in the availability and the experimental protocols that can be performed with samples from astronauts following space missions, we investigated B-cell homeostasis in healthy volunteers subjected to two months of head-down tilt bed rest, as this is the best and most integrated Earth-based analog of the microgravity of spaceflight (18). We also investigated the efficiency of dietary supplementation with a cocktail of antioxidant substances. This supplementation was chosen by the European Space Agency (ESA) because a previous study showed that it prevents lipid metabolism alterations induced by 20 days of daily steps reduction and fructose overfeeding (19). Furthermore, it prevented the decrease in the type-IIa muscle fiber cross-sectional area and was associated with lower protein ubiquitination content. The circulating antioxidant capacity was also improved following oral glucose tolerance test. Overall, our data show that bed rest seems to lower systemic inflammation, confirming the hormonal dysregulation of immunity observed in astronauts; that bed rest had no major impact on B-cell homeostasis; and that none of the assessed parameters were affected by the supplement.

## Materials And Methods

### Subjects and Ethics Statement

Twenty male volunteers were recruited for this experiment (age: 34 ± 8; height: 176 ± 5 cm; weight: 73.5 ± 6.1 kg). These subjects had no medical history or physical signs of disease. All were nonsmokers, healthy and not taking any drugs or medications. The experimental bed rest protocol, including 16 scientific protocols conducted in parallel by several research teams, assessed changes in the cardiovascular, metabolic, musculoskeletal, neurosensory, hematological and immunological systems and complied with the ethical standards of the 1964 Declaration of Helsinki. Protocols were approved (Clinical Trial.gov database number NCT03594799) by the Institutional Review Board of the “Comité de Protection des Personnes Sud Ouest et Outre Mer I” (number ID RCB: 2016-A00401–50). All subjects gave their written informed consent before they started the study.

### Overall Study Design

**Figure 1:**
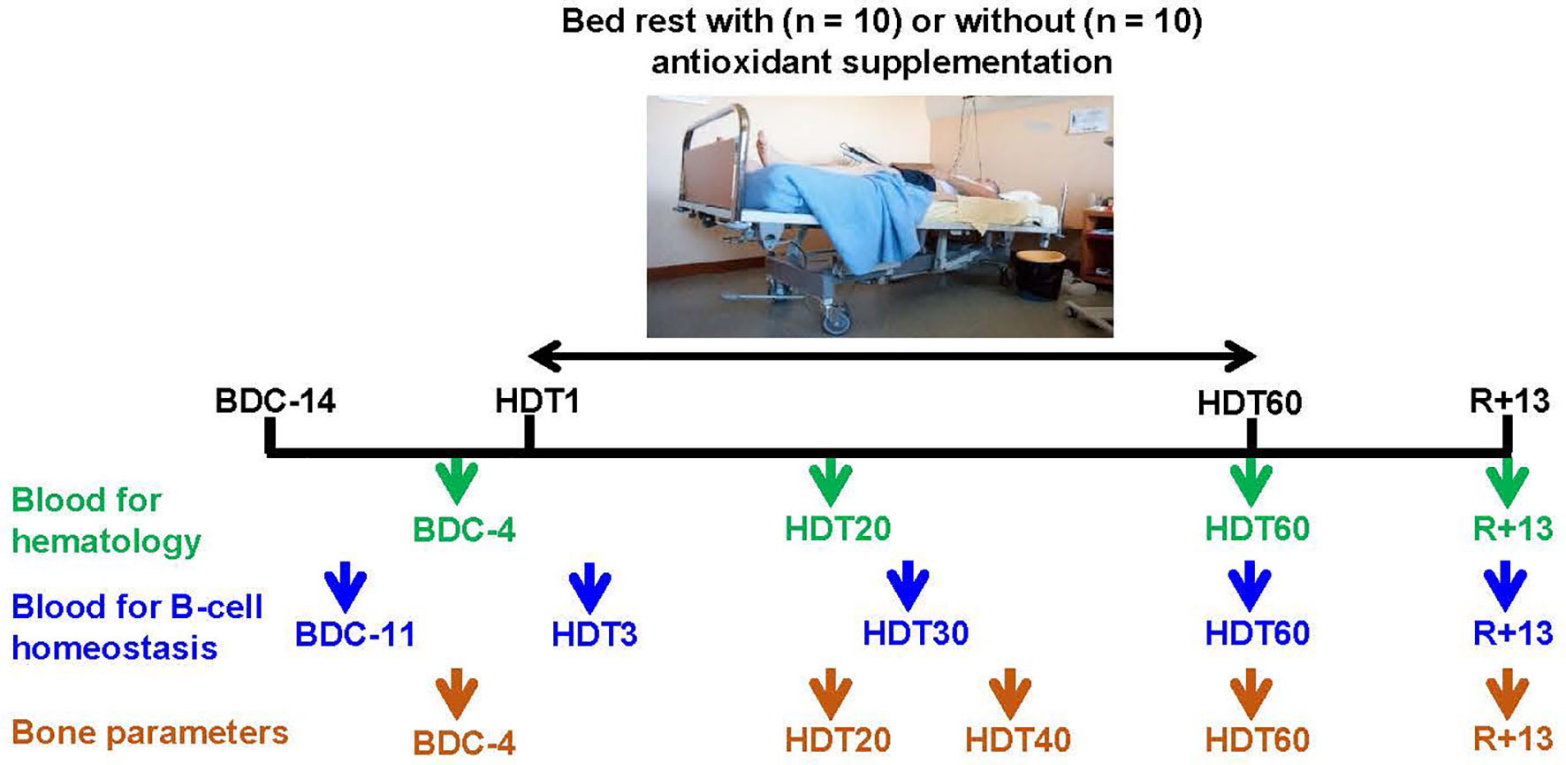
Blood and bone analysis time points. BDC, baseline data collection; HDT, head-down tilt (-6°) bed rest; R, recovery. Trial registered at clinicaltrial.gov as NCT03594799. Photograph copyright, ^©^ CNES/Barranco Rachel.

All experiments were conducted at the Space Clinic of the Institute of Space Medicine and Physiology (MEDES-IMPS) in Toulouse (France) and were coordinated by the European and French National Space Agencies (ESA and CNES, respectively). The study was divided into two campaigns (10 volunteers each). In each campaign, participants were randomly assigned to two groups in a double-blinded manner. Five of the participants were part of the control group who did not receive the antioxidant cocktail during bed rest, whereas the five others were part of the cocktail group who received the antioxidant cocktail daily during bed rest. Each campaign consisted of a 2-month head-down tilt bed rest period (HDT) with a 14-day baseline data collection period before the HDT and a 14-day recovery period after the HDT (**Figure 1**). During the HDT, the subjects laid in a supine position with a -6° tilt to preserve simulated microgravity effects. All study protocols and activities, including weighing and showering, were performed in a 6° head-down tilt position. The recovery period contained a physical rehabilitation program tailored for each volunteer.

### Antioxidant Cocktail Composition

The cocktail group received a dietary supplementation designed to prevent the metabolic alterations induced by reduced physical activity (19). The daily dose consisted of 741 mg of bioactive polyphenols (XXS-2A-BR2 mix, Spiral Company, Dijon, France), 168 mg of vitamin E coupled with 80 μg of selenium (Solgar, Marne la Vallée, France) and 2.1 g of omega-3 (Omacor, Pierre Fabre Laboratories, Toulouse, France). The 741 mg of bioactive polyphenols contained 323.4 mg of flavonols, 45.6 mg of phenylpropanoids, 78.0 mg of oligostilbenoids, 50.4 mg of coumaric acid, 135.6 mg of flavanols and 108.0 mg of flavanones. This mixture was administered as pills: two at breakfast, two at lunch and two at dinner. These polyphenols derived from Liliaceae, Vernenaceae, Lamiaceae, Vitaceae, Rubiaceae, Theaceae and Rutaceae Genres consisting of *Allium cepa*, *Lippia citriodora*, *Ajuga reptans*, *Vitis vinifera*, *Coffea robusta*, *Camellia sinensis* and *Citrus aurantium*. Vitamin E was administered as a single pill orally ingested during breakfast. The daily dose of 2.1 g of omega-3 was based on French pharmacopeia recommendations for hypolipemic effects (2-4 g/day) and is within the daily dose used in most clinical studies (20, 21). This dose, corresponding to 1.1 g of eicosapentaenoic acid (EPA) and 1 g of docosahexaenoic acid (DHA), was administrated in 3 capsules per day: one at breakfast, one at lunch and one at dinner.

### Nutrition

Based on body weight, volunteers received an individually tailored and controlled diet consistent with dietary reference intake (DRI). Energy intake differed between the phases of the study because of differences in physical activity. During the 14-day baseline data collection period, energy intake was equal to 150% of the basal metabolic rate (BMR) + 10% of the total energy expenditure (TEE). During the HDT, it was equal to 130% of BMR + 10% of TEE, and in the recovery period, it was 145% of BMR + 10% of TEE. Macronutrient intake and micronutrient intake were according to ESA bed rest standards (22) and based on DRI values (23). Protein intake was kept constant throughout the study. Energy reduction during the HDT was achieved by reducing fat and carbohydrates. All participants received one dose of vitamin D (100 000 U) one day before the initiation of the protocol, with the exception of one volunteer. Three months before the start of the study, a slight vitamin D deficiency was observed in this volunteer. Consequently, he received a first dose of vitamin D at that time and a second dose 3 months later (at HDT day 3, HDT3). Daily total fluid intake was 35-50 mL/kg/d. Volunteers were not allowed to consume coffee, tea or alcohol. Each day, participants received three meals and one afternoon snack. Menu plans and intake determinations were conducted using Nutrilog nutrition software (Nutrilog 3.11b, Nutrilog SAS, Marans, France). All meals were prepared in the metabolic kitchen at MEDES. The main dishes were provided by Davigel (Saint-Sulpice-la-Pointe, France) and prepared according to the manufacturer’s instructions. Nutrition values of the main dishes and processed foods were provided by Davigel and other industrial manufacturers and added to the Nutrilog database to ensure accurate energy and nutrient intake monitoring. Each food item was weighed and served according to volunteers’ energy needs. Leftovers were weighed and recorded, and dinner intake was adapted in accordance with breakfast and lunch leftovers. A 10-day menu cycle was applied.

### Blood Sample Collection, Red Blood Cell, Reticulocytes, Platelets, and Lymphocyte Count

Fasting venous blood was collected within 30 min after waking before other scientific protocols and several days after potentially traumatic procedures required by other participating teams (e.g., muscle biopsies) to avoid inflammation induced by these events. Red blood cells, reticulocytes, platelets, white blood cells and total lymphocytes were quantified at BDC-4, HDT20, HDT60 and R+13 (**Figure 1**) from blood samples collected in EDTA, by the LaboSud Garonne laboratory (Accreditation 31 002 325 4, Labège, France) using a SYSMEX XN9100 (SYSMEX, Roissy, France).

### Isolation of Peripheral Mononuclear Cells and Serum Collection

Fasting venous blood was collected within 30 min after waking from each volunteer at five time points (BDC-11, HDT3, HDT30, HDT60 and R+13, **Figure 1**) using two BD Vacutainer^®^ Lithium-Heparin 6 mL tubes and one BD Vacutainer^®^ SST™ II Advance 2.5 mL tube (Becton Dickinson, Franklin Lakes, NJ, USA). For peripheral mononuclear cell (PBMC) preparation, blood from the two lithium-heparin tubes was transferred into two BD Vacutainer^®^ CPT™ Mononuclear Cell Preparation Tubes with Heparin-Sodium (Ozyme, Saint-Cyr-l’Ecole, France) and centrifuged at 1500 g for 20 min at 18°C without using the brake. Once collected, PBMCs were rinsed twice in RPMI-1640 culture medium (Thermo Fisher Scientific, Waltham, MA, USA). Then, the cell pellet was resuspended in 1.5 mL of supplemented medium (RPMI-1640, 10% heat inactivated FBS, 1% penicillin-streptomycin, 1% L-glutamine) for flow cytometry analysis. For serum preparation, blood samples collected in BD Vacutainer^®^ SST™ II Advance tubes were incubated at room temperature for 30 min and then centrifuged at 2000 g for 10 min. Serum samples were collected, aliquoted and stored at -80°C until analysis.

### Flow Cytometry

A total of 0.5 x 10^6^ PBMCs were stained with the following combination of antibodies: anti-CD19-BV421 (clone HIB19), anti-CD20-PerCPCy™5.5 (clone L27), anti-CD21-PE (clone B-ly4), anti-CD27-APC (clone M-T271) and anti-CD45-V500 (clone HI30). Anti-CD19-BV421, anti-CD20-PerCPCy™5.5, anti-CD21-PE, anti-CD27-APC, anti-CD45-V500, PerCP-Cy5.5 mouse IgG1 k isotype control, BV421 mouse IgG1 k isotype control, V500 mouse IgG1 k isotype control, PE mouse IgG1 k isotype control, and APC mouse IgG1 k isotype control were purchased from BD Biosciences (Becton Dickinson, Franklin Lakes, NJ, USA). B-cell populations were identified based on the expression of the following markers: activated memory B-cells (CD19+ CD20+ CD21- CD27+ CD45+), resting memory B-cells (CD19+ CD20+ CD21+ CD27+ CD45+), tissue-like memory cells (CD19+, CD20+ CD21- CD27- CD45+), and resting naïve B-cells (CD19+ CD20+ CD21+ CD27- CD45+).

CD34+ hematopoietic progenitors in peripheral blood were enumerated using the Stem Cell Enumeration Kit (Becton Dickinson).

All flow cytometry analyses were performed using a BD LSRFortessa™ Flow Cytometer (Becton Dickinson) from the I2MC cytometry platform (CHU Rangueil, Toulouse, France) and BD FACSDiva™ Software (Becton Dickinson).

### Cortisone and Cortisol Quantification

Plasma samples (100 µL) spiked with 1 ng cortisol-d4 (CIL, Andover, USA) and 1 ng cortisone-d8 (Sigma-Aldrich, Taufkirchen, Germany) as internal standards were extracted with 1 mL methyl *tert*-butyl ether. After evaporation of the ether phase under vacuum, the dried contents were reconstituted with 16 µL acetonitrile and 16 µL mobile phase A (0.1% acetic acid (v/v) in water/acetonitrile (95:5, v/v)). High-performance liquid chromatography separation was performed using an Agilent 1290 system with a Kinetex C18 column (50 × 2.1 mm x 2.6 μm; Phenomenex, Aschaffenburg, Germany). The column oven temperature was set to 30°C, and 5 µL of reconstituted sample was injected. Mobile phase B consisted of 0.1% acetic acid (v/v) in water/acetonitrile (5:95, v/v). After a 2.5 min equilibration of the column with 90% A/10% B at a flow rate of 0.3 mL/min, a 1 min period at 90% A/10% B and a flow rate of 0.3 mL/min followed. The gradient then was hold for 9 min at 0% A/100% B and a flow rate of 0.5 mL/min. After another 2.5 min step at 0% A/100% B at 0.5 mL/min, the gradient was increased to 90% A/10% B for another 0.5 min at 0.3 mL/min flow. Mass spectrometric analysis was performed with a hybrid quadrupole time-of-flight (ToF) mass spectrometer (TripleTOF^®^ 6600, AB Sciex, Darmstadt, Germany). Ion source temperature was 600°C, the ion spray voltage was set to 5500 eV and collision energy was adjusted to 35 eV. Mass spectra were obtained in positive ionization mode over a scan range of 180 - 400 Da. Analyst TF^®^ 1.7.1 and MultiQuantTM 3.0 software were used for data acquisition and analysis (AB Sciex, Darmstadt, Germany).

### Quantification of Serum Cytokines and Antibody Isotypes

Tumor necrosis factor alpha (TNFα), interleukin (IL)-1β, IL-1 receptor antagonist (IL-1RA), IL-6, IL-10 and IL-12p40 levels in serum samples were analyzed using a customized multiplex magnetic bead array (HCYTOMAG-60K-07, Merck, Darmstadt, Germany). Serum IgG1, IgG2, IgG3, IgG4, IgA and IgM were quantified using the MILLIPLEX^®^ MAP Human Isotyping Magnetic Bead Panel, while serum IgE was quantified using the MILLIPLEX^®^ MAP Human Immunoglobulin IgE Single Plex Magnetic Bead Kit (Merck). In all cases, assays were performed according to the manufacturer’s instructions. Samples were run on a MAGPIX instrument (Luminex, Merck) and analyzed with MILLIPEX analyst standard version 5.1 (Merck).

### Bone Parameters

Left tibia bone density and microarchitecture were studied at BDC-4, HDT20, HDT40, HDT60 and R+13 (**Figure 1**) using noninvasive XtremeCT technology (3DpQCT, Scanco Medical, Brüttisellen, Switzerland) at the Rangueil University Hospital in Toulouse, France.

### Statistics

Data collected during bed rest were first analyzed for the statistical significance of the effects of dietary supplementation with either a repeated-measures two-way ANOVA with the Geisser-Greenhouse correction in combination with *post hoc* Sidak’s multiple comparison testing or, when data points were missing, with a Geisser-Greenhouse corrected linear mixed effects model using the restricted maximum likelihood method in combination with *post hoc* Sidak’s multiple comparison testing (GraphPad Prism 9.0). As no statistical significance was identified between cocktail and control volunteers, pooled data for all volunteers per time point were analyzed for the statistical significance of the effects of bed rest and test days using either a repeated-measures one-way ANOVA with the Geisser-Greenhouse correction in combination with *post hoc* Tukey multiple comparison testing or, when data points were missing, with a Geisser-Greenhouse corrected linear mixed effects model using the restricted maximum likelihood method in combination with *post hoc* Tukey multiple comparison testing (GraphPad Prism 9.0). More complex models (nested random effects, random slopes, etc.) did not converge. Outliers were identified and removed using the Grubbs’ test (α = 0.05). Information on the age, height and weight of the test subjects was not considered because these parameters were not different between the 2 groups (cocktail *vs.* control). An adjusted P value < 0.05 was considered statistically significant.

## Results

All participants completed the 60 days of bed rest. The cocktail and control groups were similar in terms of age, height and weight. None of the parameters analyzed in this study were significantly affected by antioxidant supplementation, as revealed by either two-way ANOVA or mixed effects analyses. As a consequence, individuals of both groups were pooled.

### Reticulocytes, Erythrocytes and Platelets

**Figure 2:**
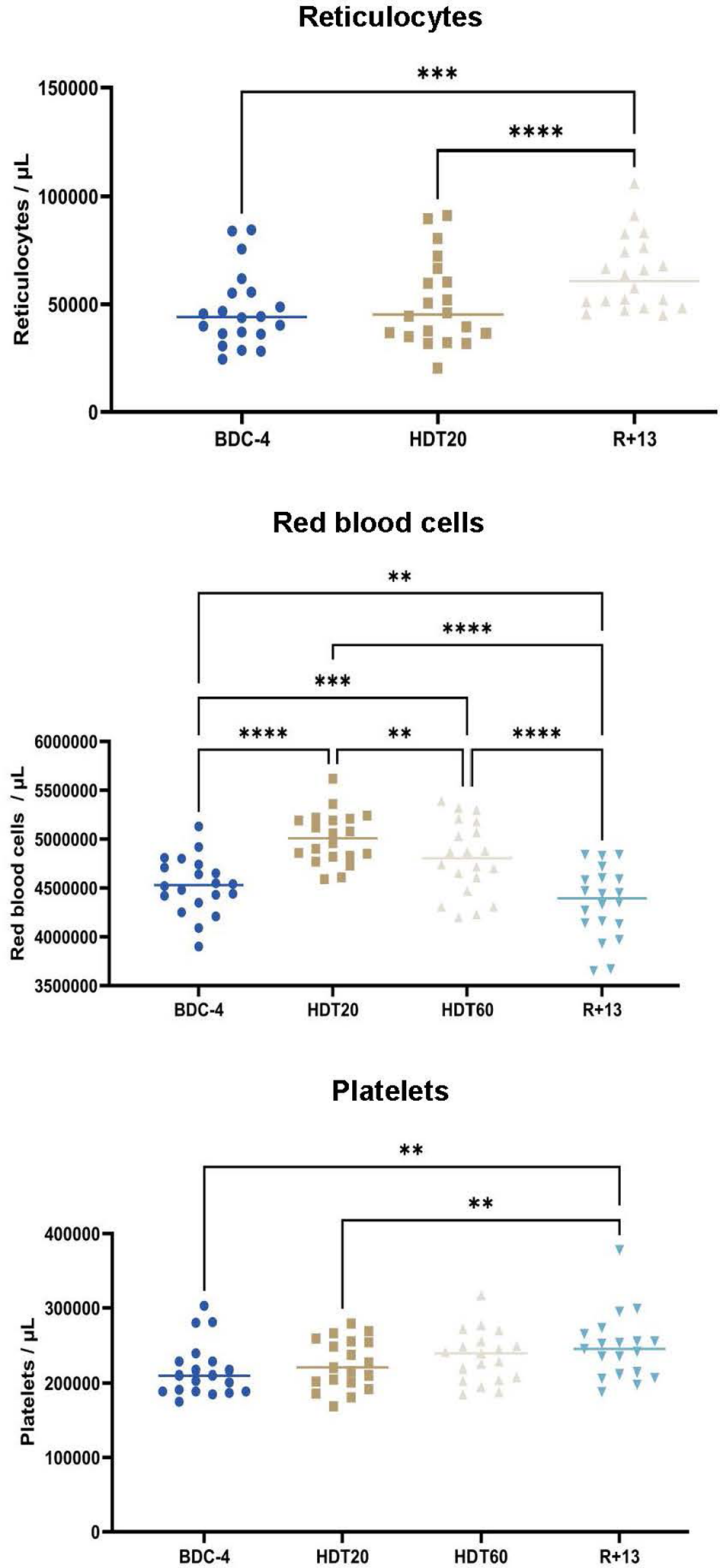
Evolution of reticulocytes (n = 20), erythrocytes (n = 20) and platelets (n = 19) in the blood of volunteers before (BDC), during (HDT) and 13 days (R+13) after the end of bed rest. As no statistically significant differences between the control and cocktail groups were observed, individuals of both groups were pooled. Horizontal bars indicate the median. Statistically significant differences were revealed using either one-way ANOVA or a linear mixed effects model analysis. **p < 0.01; ***p < 0.001, ****p < 0.0001.

We studied the effects of this trial at BDC-4, HDT20, HDT60 and R+13 on reticulocytes, erythrocytes and platelets as these cell types have been shown to be affected by bed rest (24–26), thereby indicating a modification of hematopoiesis which includes B-lymphopoiesis. Reticulocytes could not be analyzed at HDT+60, but **Figure 2** indicates that they were more abundant at R+13 than at BDC-4 (+ 37 ± 47% (median ± SD), p<0.001) and HDT20 (+ 28 ± 34%, p<0.0001). Red blood cells increased after 20 days of 6° head-down tilt bed rest (+ 10 ± 5% by comparison to BDC-4, p<0.0001). Then, the number of red blood cells gradually decreased to reach a mean value lower than baseline (- 6 ± 5% at R+13 in comparison to BDC-4, p<0.01). Platelets, similarly to reticulocytes, were more abundant at R+13 than at BDC-4 (+ 15 ± 12%, p<0.01) and HDT20 (+7 ± 10%, p<0.01).

### B-Cell Homeostasis

**Figure 3:**
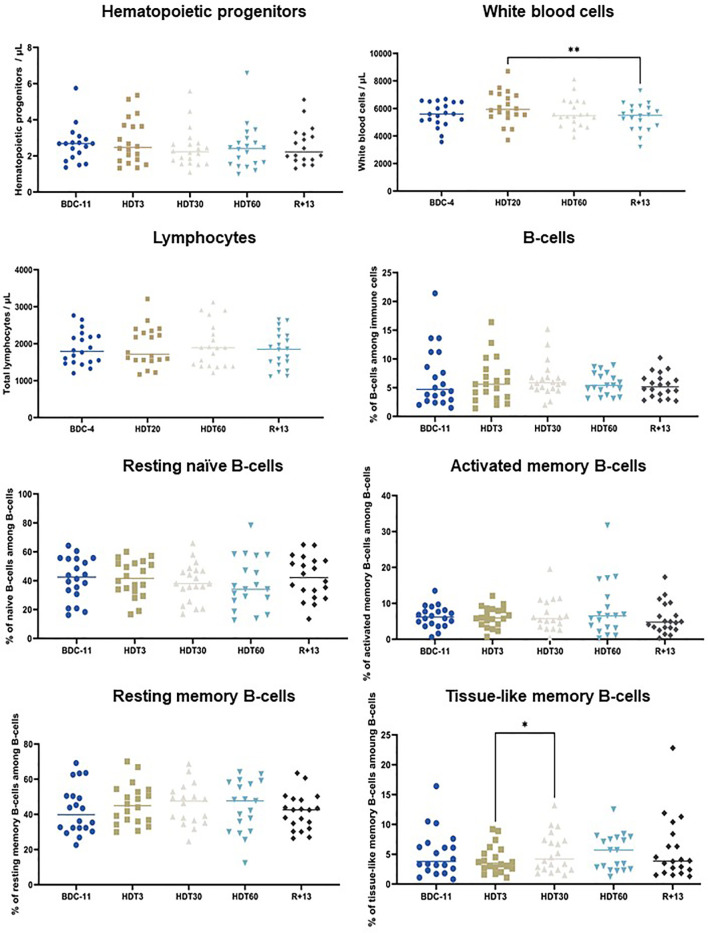
Effects of bed rest on hematopoietic progenitors, white blood cells, circulating lymphocytes, total B-cells, resting naïve B-cells, resting memory B-cells, activated memory B-cells and tissue-like memory B-cells. As no statistically significant differences between the control and cocktail groups were observed, individuals of both groups were pooled. n = 18-20. Horizontal bars indicate the median. Statistically significant differences were found using either one-way ANOVA or a linear mixed effects model analysis. *p < 0.05; **p < 0.01. BDC, baseline data collection; HDT, head-down tilt bed rest; R, recovery.

We then studied the effects of this bed rest protocol on hematopoietic progenitors, white blood cells, total circulating lymphocytes, total circulating B-cells and four B-cell subpopulations (resting naïve B-cells, activated memory B-cells, resting memory B-cells and tissue-like memory B-cells). **Figure 3** shows that white blood cells were significantly increased at HDT20 compared to R+13 (+ 7 ± 13%, p<0.01), but this increase was within physiological limits (4000/µL to 9000/µL) and was not present at HDT60. The percentage of tissue-like memory B-cells was significantly increased at HDT30 compared to HDT3 (p<0.05), but this difference disappeared at HDT60 and R+13. No significant changes could be noted for other populations. Taken together, these data show that B-cell homeostasis was maintained during this long, 60-day period of bed rest.

### Antibody Isotypes

**Figure 4:**
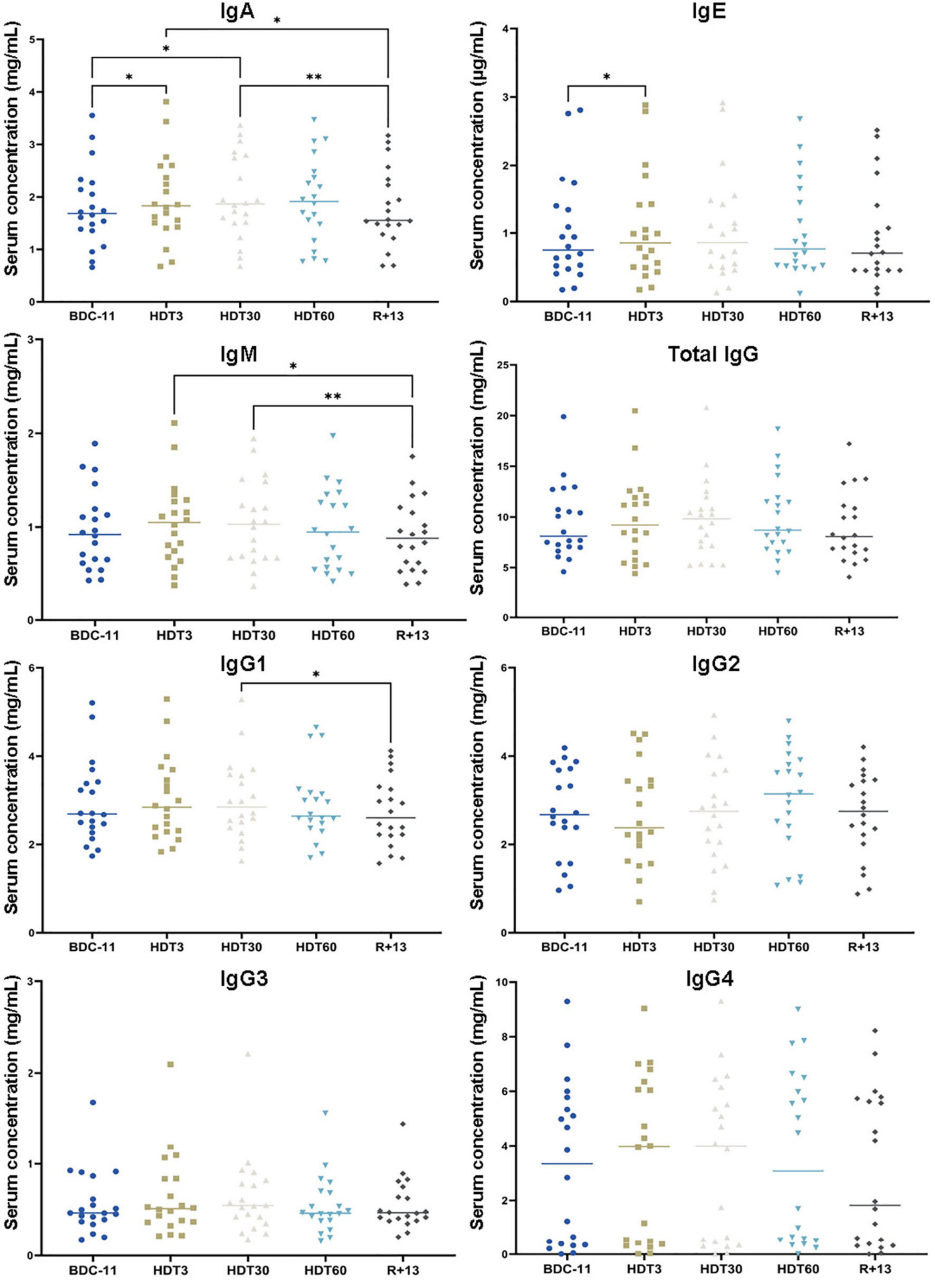
Changes in serum isotype concentrations. As no statistically significant differences between the control and cocktail groups were observed, individuals of both groups were pooled. n = 20. Horizontal bars indicate the median. Statistically significant differences were found using either one-way ANOVA or a linear mixed effects model analysis. *p < 0.05; **p < 0.01. BDC, baseline data collection; HDT, head-down tilt bed rest; R, recovery.

We also quantified serum IgA, IgE, IgM, total IgG, IgG1, IgG2, IgG3 and IgG4. **Figure 4** shows that IgA concentration increased during bed rest (+ 10 ± 13% at HDT3 by comparison to BDC-11, p<0.05; + 11 ± 13% at HDT30 by comparison to BDC-11, p<0.05) and returned to a value close to baseline at R+13. IgE were more abundant at HDT3 than at BDC-11 (+ 5 ± 7%, p<0.05). IgM were less abundant at R+13 by comparison to values observed during bed rest (- 17 ± 14% at R+13 by comparison to HDT3, p<0.05, and HDT30, p<0.01). Finally, we noted that total IgG were not affected by bed rest, nor were IgG2, IgG3 and IgG4, despite a higher IgG1 concentration at HDT30 by comparison to R+13 (+ 7 ± 14%, p<0.05).

### Bone Density and Microarchitecture

**Figure 5:**
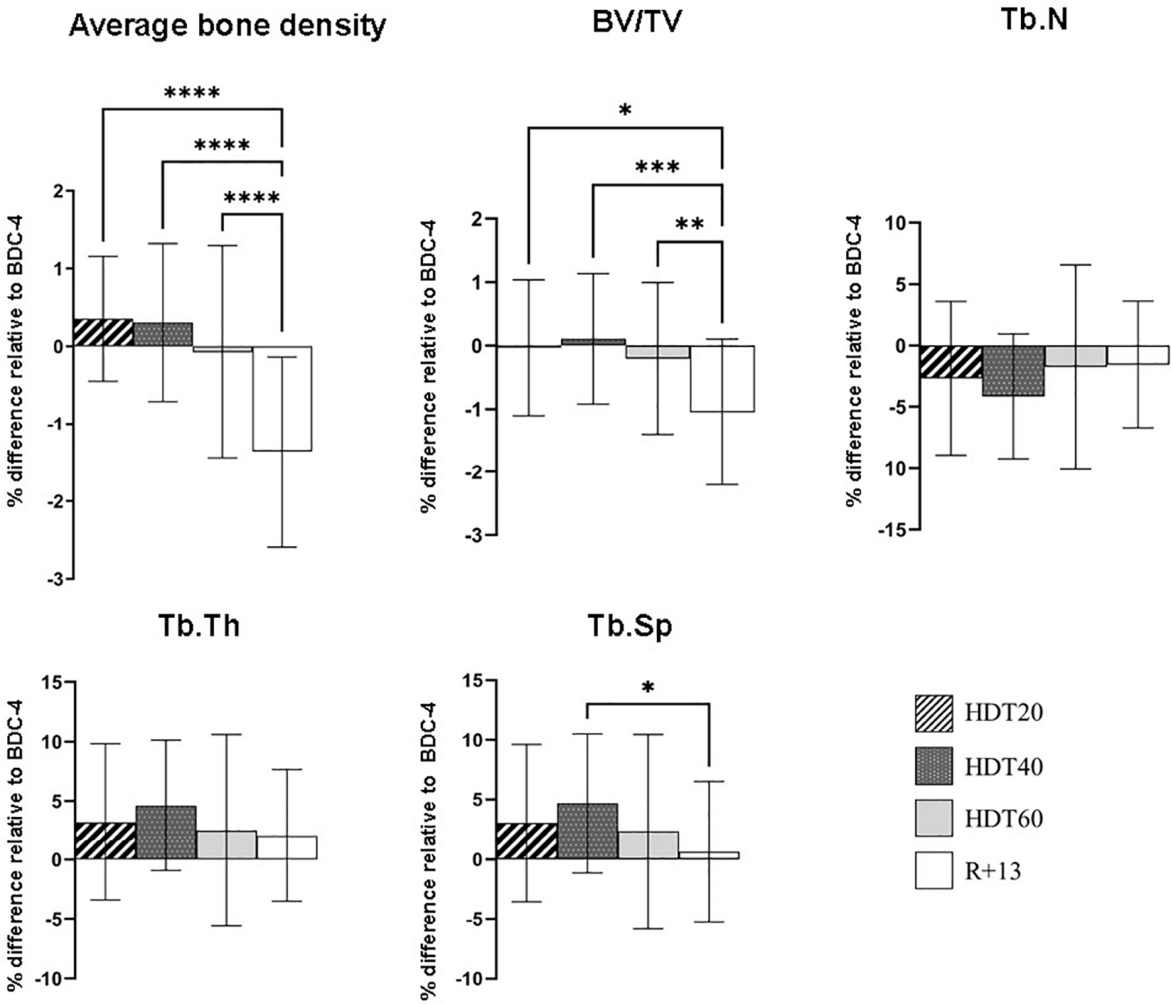
Changes in left tibia bone parameters during bed rest. As no statistically significant differences between the control and cocktail groups were observed, individuals of both groups were pooled. n = 20. Values are expressed as the percentage of difference relative to BDC-4 ± SD. Statistically significant differences were revealed using either one-way ANOVA or a linear mixed effects model analysis. *p < 0.05; **p < 0.01; ***p < 0.001, ****p < 0.0001. BV/TV, bone volume fraction; Tb.N, trabeculae number; Tb.Th trabecular thickness; Tb.Sp trabecular separation.

Given that B lymphocytes are derived from hematopoietic stem cells (HSCs) residing in the bone marrow in specialized niches made up of bone and vascular structures, and that previous reports indicated that bed rest induces bone loss (27–30), we analyzed tibial bone mineral density and microarchitecture. Significant decreases in average bone density (- 1.4 ± 1.2%, p<0.0001) and bone volume fraction (BV/TV) (- 1 ± 1.1%, p<0.05-0.001) were noted at R+13 (**Figure 5**). Trabeculae number (Tb.N) and thickness (Tb.Th) were not significantly affected, while trabecular separation (Tb.Sp) increased during bed rest (+ 4.6 ± 5.5% at HDT40, p<0.05) and returned close to the baseline value at R+13. These data show that bed rest induced changes in bone morphometric parameters, but these results did not explain the lack of an impact of bed rest on circulating progenitors and B cell homeostasis.

### Serum Cytokine Levels

**Figure 6:**
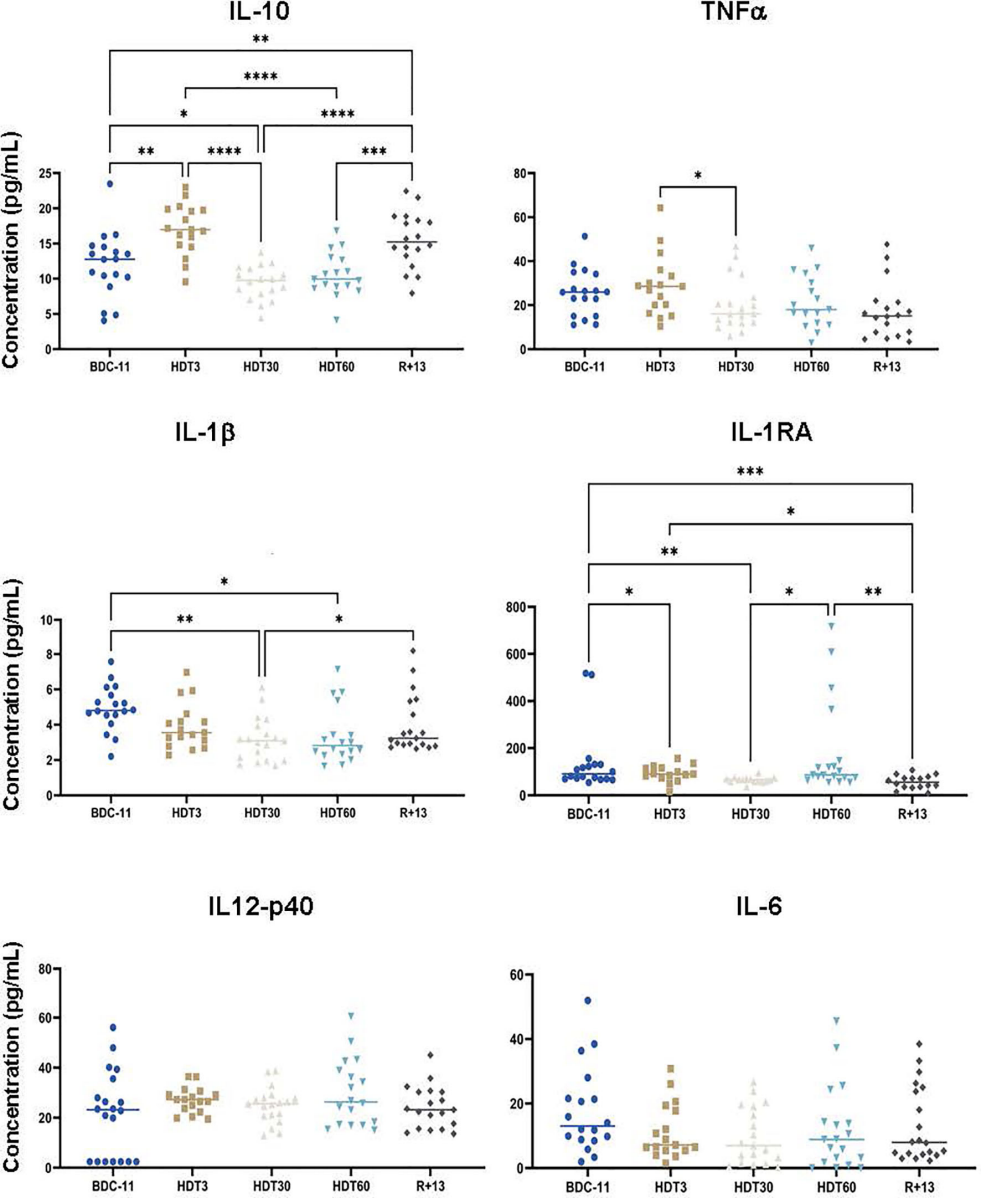
Mean concentrations of serum pro- and anti-inflammatory cytokines before (BDC), during (HDT) and after (R) bed rest. As no statistically significant differences between the control and cocktail groups were observed, individuals of both groups were pooled. n = 17-20 except for IL-1RA where n = 15-19. Horizontal bars indicate the median. Statistically significant differences were shown using either one-way ANOVA or a linear mixed effects model analysis. *p < 0.05; **p < 0.01; ***p < 0.001, ****p < 0.0001. Assay sensitivities (pg/ml): IL-10, 1.1; TNFα, 0.7; IL-1β, 0.8; IL-1RA, 8.3; IL-12p40, 7.4; IL-6, 0.9.

As inflight cytokine dysregulation was previously reported in astronauts and cosmonauts involved in a long-duration spaceflight onboard the ISS (31, 32), we quantified six pro- and anti-inflammatory cytokines in serum samples collected before, during and after bed rest. These analyses (**Figure 6**) revealed that IL-10 concentration increased at HDT3 (+ 36 ± 65% in comparison to BDC-11, p<0.01), decreased below the baseline value at HDT30 (- 21 ± 24% in comparison to BDC-11, p<0.05) and increased again at R+13 (+ 34 ± 55% in comparison to BDC-11, p<0.01). The TNFα concentration was 34 ± 27% lower at HDT30 than at HDT3 (p<0.05). In the same way, the amount of IL-1β was decreased at HDT30 and HDT60 (- 45 ± 34%, p<0.01 and - 48 ± 36%, p<0.05 in comparison to BDC-11, respectively). The concentration of IL1-RA, which inhibits IL-1β action, differed on an individual basis. Finally, the IL-6 and IL12p40 concentrations did not change during bed rest. These data suggest a lower systemic inflammatory status during bed rest.

### Cortisone and Cortisol

**Figure 7:**
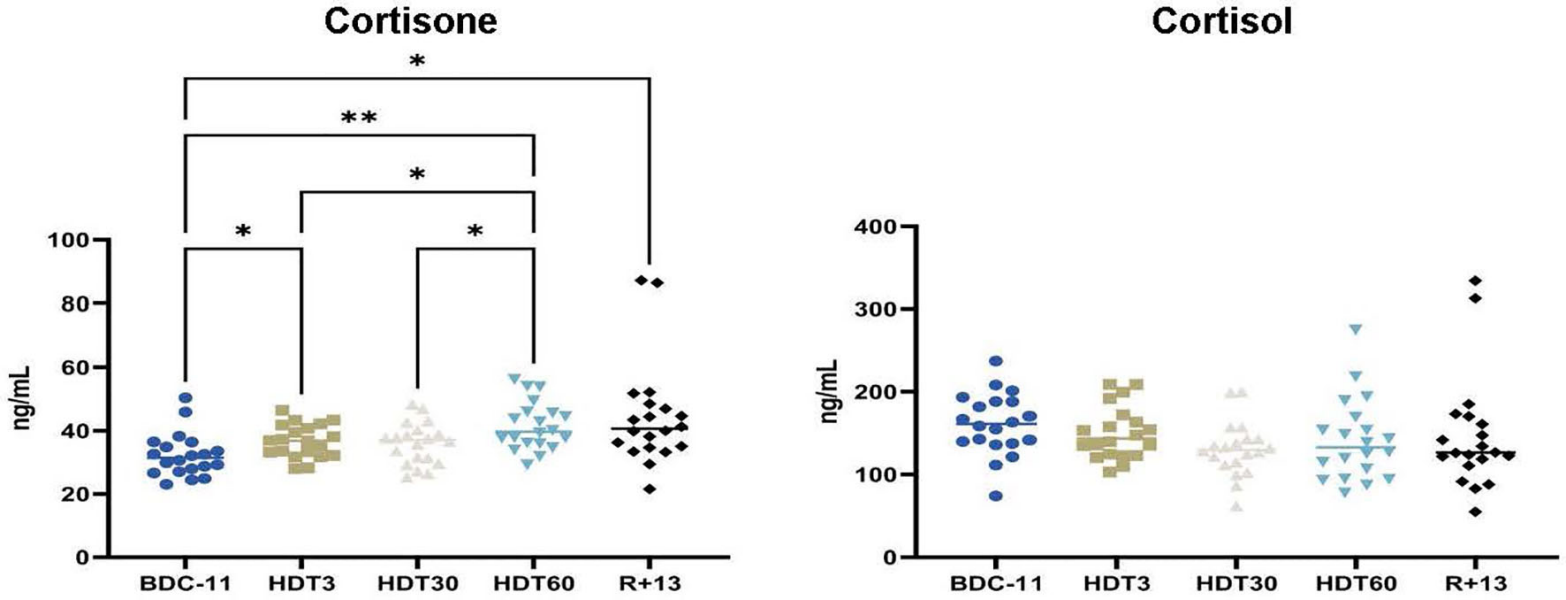
Kinetics of serum cortisone and cortisol levels. As no statistically significant differences between the control and cocktail groups were observed, individuals of both groups were pooled. n = 20 except for cortisone at R+13 where n = 18. Horizontal bars indicate the median. Statistically significant differences were found using either one-way ANOVA or a linear mixed effects model analysis. *p < 0.05; **p < 0.01.

Finally, we quantified cortisone, a well-known anti-inflammatory hormone, and cortisol, a major stress hormone made by the 11β-hydroxysteroid dehydrogenase 1-mediated reduction of cortisone. Our results show that serum cortisone concentrations gradually increased during bed rest (+ 32 ± 35% at HDT60 in comparison to BDC-11, p<0.01), while cortisol concentrations were constant throughout the trial and close to the levels observed in unstressed individuals (50-200 ng/mL at 08:00 AM) (**Figure 7**). This gradual increase in cortisone concentration agrees with the lower inflammatory status revealed *via* the quantification of pro- and anti-inflammatory cytokines.

## Discussion

### Antioxidant Supplementation Does Not Affect Bed Rest-Induced Changes

This investigation was performed under highly controlled conditions and guaranteed that the volunteers remained in a metabolic ward with a strictly controlled environment and monitoring during the entire study period. This standardization made it possible to control and adapt energy and nutrient intakes to the needs of each subject. This study revealed that supplementation with a cocktail of antioxidants (polyphenols, vitamin E, selenium and omega-3) had no effects on B-cell homeostasis, serum antibody isotype concentrations, hematological, bone, cytokine and stress parameters studied here. In the same way, other studies carried out independently on the same subjects did not show any effect of this supplementation on biomarkers of calcium homeostasis, bone formation and resorption (33); lumbar vertebral fat fraction (34); hemolysis and CO elimination (35); muscle deconditioning, oxidative damage, mitochondrial content and protein balance (36); neurobehavior (37); or cardiac circadian rhythm (38). This lack of an effect of the supplement was not expected, as polyphenols, vitamin E, selenium and omega-3 are known to have positive effects on numerous biological systems (39–48). The most likely explanation is that, as indicated by Austermann et al. (33), all volunteers were adequately supplied with energy, macro- and micronutrients. Volunteers received an individually tailored and controlled diet consistent with dietary intake references, and such a diet already contains between 500 and 1500 mg/d of antioxidants (49). Thus, diets of the cocktail and control groups likely contained sufficient amounts of antioxidants so that the supplement might not have induced a further benefit.

### Red Blood Cells, Reticulocytes, and Platelets Confirm That Bed Rest Is a Well-Adapted Earth-Based Analog

Our analyses revealed that red blood cell concentration was significantly elevated at HDT20 (+ 10 ± 5%) and HDT60 (+ 5 ± 6%) and returned to almost the baseline value at R+13. A similar profile was observed in a previous bed rest study (24), and a similar increase in red blood cell count (+ 9%) was observed in astronauts throughout a long-duration spaceflight (50), in which case, similar to this study, the red blood cell concentration returned slightly below baseline after landing. In these three cases, this return to a value close to baseline could be due to plasma volume shifts associated to saline drip upon reentry into gravity (51, 52) and body fluid redistribution when volunteers switch from a prone to an upright position. Regarding reticulocytes, as noted here, Liu et al. (34) reported an increase in their number after bed rest, while this number was not significantly modified during bed rest exposure. These authors demonstrated that this observation was due to an enhancement of erythropoiesis during recovery coupled with the consumption of fatty acids stored in regulated marrow adipocytes as energy for erythroblasts. Another recent study showed a great decrease in reticulocytes in astronaut peripheral blood on the first day after landing followed by a rapid increase to reach, 10 days after landing, levels above those determined before the flight (53). Unfortunately, as reticulocytes were not quantified at HDT60 and R+1, we could not observe such phenomena, but the rapid increase during the first 10 days after landing noted by Cao et al. (53) supports enhanced erythropoiesis. Concerning platelets, a recent study indicated, contrary to what was thought before (54), that long-duration spaceflight does not induce thrombocytopenia (50). Similarly, it was shown that platelet count did not change during a 60-day bed rest (26). These observations agree with our data showing that platelet concentration remained stable during this two-month bed rest. The increase noted at R+13 could be due to a slight increase in thrombopoiesis in parallel to that of erythropoiesis, as both occur at the same time point, but this hypothesis warrants further investigation. These similarities between space and bed rest hematological data confirm that bed rest is a well-adapted Earth-based analog of the microgravity of spaceflight (18) and underlines the validity of the methodological approach.

### Two Months of Head-Down Tilt Bed Rest Do Not Affect B-Cell Homeostasis

We studied the effects of bed rest on hematopoietic progenitors, white blood cells, total circulating lymphocytes, total circulating B-cells and four B-cell subpopulations. Hoff et al. (55) observed an increase in progenitors after 60 days of bed rest and attributed this increase to bone marrow adiposity, which would alter stem cell niches, leading to increased release of progenitors into peripheral blood. Similarly, we observed an egress of hematopoietic cells out of the bone marrow in mice subjected to 21 days of AOS, suggesting that simulated microgravity impairs interactions with the endosteal niche (15). However, during this bed rest study, the lumbar vertebral fat fraction was unchanged from baseline (34), and no change in the abundance of progenitors in the bloodstream was observed, suggesting that the bone marrow microenvironment was not affected (see also below). Although the literature may vary, the common finding among this study and previous bed rest studies appears to be unaltered numbers of white blood cells, total lymphocytes and total B-cells (56–60). The analysis of 23 astronauts and 12 cosmonauts participating in long-duration expeditions aboard the ISS revealed an elevated white blood cell count during flight, which was aggravated just after landing, due to increased neutrophil counts (32, 61). In this bed rest study, a slight increase in neutrophils was only noted at R+1 (33), but it did not result in a significant increase in white blood cells at R+13. These increases in neutrophils are commonly attributed to stress-induced demargination. The absence of a significant increase in serum cortisol concentration during bed rest likely explains the limited change in neutrophils and consequently white blood cells. Analyses performed on the same 23 astronauts revealed that total circulating lymphocytes and B-cells were unaltered during flight (61). The same observation was recently made in 6 astronauts who participated in 13-15-day space missions (53). However, increases in total circulating lymphocytes and B-cells were noted at the end of the mission and directly following landing in 12 cosmonauts (32). These discrepancies may be related to methodological differences and subject demographics. The analysis of four B-cell subpopulations (resting naïve B-cells, activated memory B-cells, resting memory B-cells and tissue-like memory B-cells) strongly suggested that B-cell homeostasis was maintained during this long-duration bed rest study. A modification of tissue-like memory B-cells was noted between HDT3 and HDT30, but this difference may well be because this cell type is not abundant, so a small change in the number of these cells can be statistically significant. A slight increase (~10%) in memory B-cells was also previously noted in 8 Chinese volunteers involved in a 45-day bed rest study (62). However, in agreement with our data, no effect of spaceflight on the different B-cell subsets was noted in 8 crewmembers who completed a 6-month stay on the ISS (17). These authors also showed that IgG and IgM remained unchanged during and after spaceflight, while plasma IgA concentrations were elevated inflight compared with baseline and recovery values. Similarly, IgA increased while total IgG and IgM concentrations did not change during bed rest. However, contrary to Spielmann et al. (17), a 17% ± 14% decrease of IgM was noted at R+13. An increase of IgE was noted after 3 days of bed rest. Since this increase was limited (+ 5% ± 7%) and not associated to an increase of eosinophils (**Supplementary Figure 1**) we can rule out an allergy.

The lack of an effect of bed rest on circulating progenitors and B-cells, in contrast to mice subjected to AOS (15, 63), could be explained, at least in part, by the fact that bed-rest-related bone changes, which are comparable to those from previous bed rest studies (30), are less severe than those induced by 21 days of AOS (- 20% and - 30% decreases of tibia Tb.N and BV/TV, respectively, in mice after 21 days of AOS) (15). However, it is possible that during future deep-space exploration missions that will be of unprecedented durations, bone loss will reach a level inducing a reduction in B lymphopoiesis and consequently of B-cells in the periphery, as observed in mice flown for one month onboard the BION-M1 biosatellite (16). Indeed, bone changes are often more rapid during space journeys than with bed rest (30, 64). Further studies will be needed to address this important question.

### Potential Reduction of the Systemic Inflammatory Status During Bed Rest

Low levels of serum cytokines, except for IL-1RA, which is constitutively present [IL-1RA is constantly approximately 100-fold higher than IL-1 (65)] were noted in this study, consistent with healthy volunteers free of infection. This observation also suggests that studies conducted by other scientific teams participating to this large-scale project had minimal effects on our results. An overexpression of IL-10 (+ 36 ± 65%), a major anti-inflammatory cytokine, was observed at the beginning of bed rest, followed by decreases in two pro-inflammatory cytokines (- 45 ± 34 to - 48 ± 37% of IL-1β and -34 ± 27% of TNFα), suggesting a potential lower systemic inflammatory status during bed rest, supported by an increase in serum cortisone which has anti-inflammatory properties. This lower inflammatory status cannot be attributed to the supplement because, as indicated above, no statistically significant differences could be observed between control and cocktail subjects. In the same way, a time-related progressive increase in soluble TNFα receptor levels, which binds and neutralizes TNFα and therefore has an anti-inflammatory role, was noted during a previous 60-day bed rest study involving female volunteers (66), and mice subjected to a model used to mimic socioenvironmental stresses encountered during spaceflight (not considering high-stress phases such as extravehicular activities or docking/undocking) tended to show reduced levels of serum proinflammatory cytokines (67). In contrast, the analysis of plasma samples collected inflight during relatively lower-stress mission phases (away from vehicle dockings and extravehicular activities) in 28 astronauts participating in long-duration ISS mission revealed persistent low-grade systemic inflammation characterized by increased TNFα and IL-1RA plasma concentrations (31). This increase in proinflammatory cytokines was confirmed in cosmonauts participating in long-duration missions (>140 days) (32) and by a recent study of 14 previously uninvestigated cytokines (68). Interestingly, one of these three studies (32) also quantified cortisol and showed that the levels of this molecule were not altered. This lack of cortisol increase could perhaps explain the increase in proinflammatory cytokines. However, very interestingly, when the peripheral blood mononuclear cells of 23 astronauts involved in a long-duration mission on the ISS were collected inflight and stimulated with PMA and ionomycin, a strong mitogen stimulus activating most leukocyte populations, inflammatory cytokine concentrations in culture supernatants were lower than those obtained 180 days before launch (61), suggesting that inflight-mounted inflammatory responses could be reduced. Thus, the modulation of inflammation, which is the immune system’s response to harmful stimuli and is a vital defense mechanism, likely contributes to explaining why nearly half of astronauts spending 6 months onboard the ISS face immunological problems (13). This modulation of inflammation is multifactorial because both bed rest used to simulate microgravity and the model used to mimic socioenvironmental stresses encountered inflight affect basal levels of cytokines involved in this biological process and is also observed in lower vertebrates in response to stressors similar to those encountered during a stay on the ISS (69).

### Limitations

This study is limited by the lack of a placebo due to the characteristic taste of the fish oil capsules used and difficulties in finding a matching placebo, the fact that data could not be adjusted for plasma volume, the limited duration of bed rest exposure, and the absence of space radiation. However, we are confident in our results because the subjects were under highly controlled study conditions and, as shown above, our data are consistent with previously collected space data.

## Conclusions

In summary, this work shows that 2 months of bed rest seems to lower the systemic inflammatory status but does not affect B-cell homeostasis, as previously shown in astronauts embarked on a 6-month stay in the ISS. However, these results do not presage the effects of future deep-space exploration missions, which will be of unprecedented durations, on B-cell homeostasis and on the quality of the humoral immune responses that could be developed in response to an antigen. Indeed, animal studies have shown that although similar quantities of antibodies could be produced in response to an antigenic challenge during a 5-month spaceflight (5), the quality of the produced antibodies was inferior to that of those produced on Earth (6). Immunization and B-cell homeostasis analysis during long-term missions (> 1 year) will have to be performed to shed light on these important questions. Finally, we show that the applied nutritional countermeasure was not effective in preventing bed rest-induced changes likely because volunteers of the control and cocktail groups were fed a controlled diet consistent with dietary reference intake.

## Data Availability Statement

The raw data supporting the conclusions of this article will be made available by the authors, without undue reservation.

## Ethics Statement

The studies involving human participants were reviewed and approved by Institutional Review Board of the “Comité de Protection des Personnes Sud Ouest et Outre Mer I”. The patients/participants provided their written informed consent to participate in this study.

## Author Contributions

JB, BB, DM, SG, SM, ER, RV, and AK performed the experiments and analyzed the data. JB and BB performed statistical analyses. JB, BB, and J-PF wrote the manuscript. DM, SG, SM, ER, RV, AK, SB, and AC revised the manuscript. J-PF designed and supervised the project. SB, AC, and J-PF obtained funding resources. All authors contributed to the article and approved the submitted version.

## Funding

This bed rest study was funded by the European Space Agency and the Centre National d’Etudes Spatiales (CNES, the French Space Agency). Investigators’ costs were covered by CNES (grants DAR 4800000841, 4800000894 and 4800000950), the French Ministry of Higher Education and Research, the Université de Lorraine, the French State-Region Project Contract (CPER), DLR on behalf of the Federal Ministry of Economics and Technology/Energy (grants 50WB1931 and 50WB1622), and by the ESA/BELSPO/Prodex IMPULSE contract (CO-90-11-2801-03 and 4000109861).

## Conflict of Interest

The authors declare that the research was conducted in the absence of any commercial or financial relationships that could be construed as a potential conflict of interest.

## Publisher’s Note

All claims expressed in this article are solely those of the authors and do not necessarily represent those of their affiliated organizations, or those of the publisher, the editors and the reviewers. Any product that may be evaluated in this article, or claim that may be made by its manufacturer, is not guaranteed or endorsed by the publisher.
