# Supplementary material for: B-Cell Homeostasis Is Maintained During Two Months of Head-Down Tilt Bed Rest With or Without Antioxidant Supplementation
Source: Front Immunol. 2022 Feb 16;13:830662. doi: 10.3389/fimmu.2022.830662 (PMC8892569; doi:10.3389/fimmu.2022.830662)
Supplement: Supplementary file 1 [file Image_1.pdf]

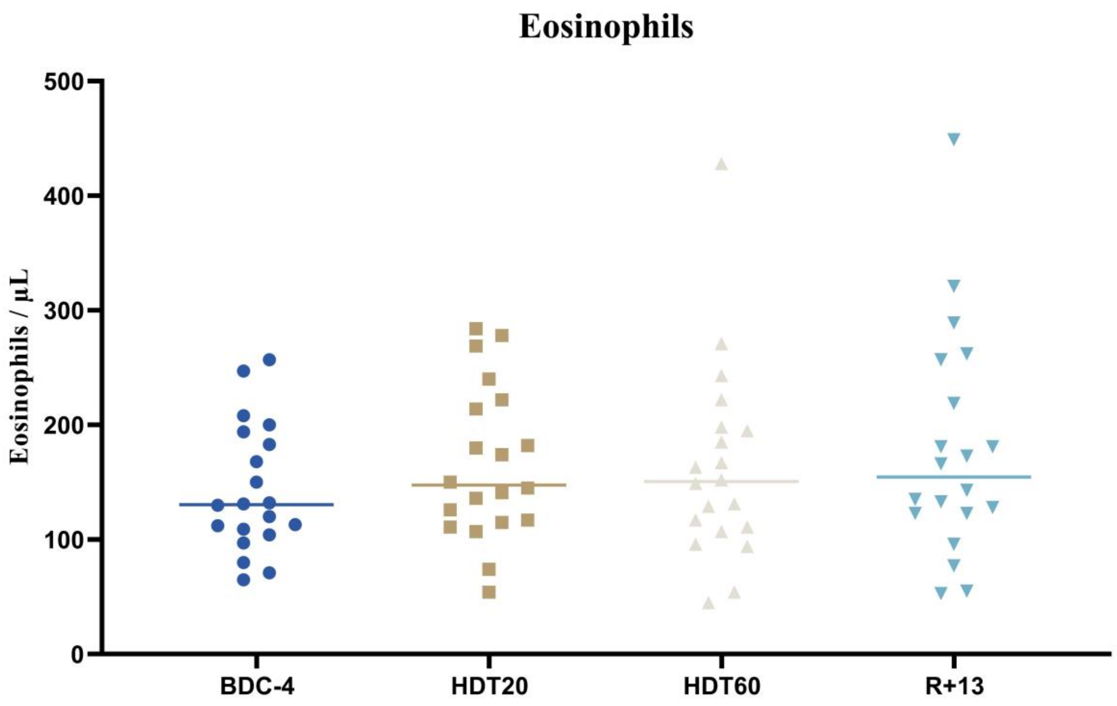

Supplementary Figure 1. Effect of bed rest on eosinophil count. Eosinophils were quantified at BDC-4, HDT20, HDT60 and R+13 from fasting blood samples collected within 30 min after waking, in EDTA, by the LaboSud Garonne laboratory (Accreditation 31 002 325 4, Labège, France) using a SYSMEX XN9100 (SYSMEX, Roissy, France). As no statistically significant differences between the control and cocktail groups were observed, individuals of both groups were pooled. n = 20. Horizontal bars indicate the median. No statistically significant differences were found using either one-way ANOVA or a linear mixed effects model analysis. BDC, baseline data collection; HDT, head-down tilt bed rest; R, recovery.
